# Extensibility of autologous pericardium roll conduit in non-confluent pulmonary artery: a case report

**DOI:** 10.1186/s13019-019-0913-z

**Published:** 2019-06-03

**Authors:** Fumiya Yoneyama, Toru Okamura, Yorikazu Harada

**Affiliations:** Department of Cardiovascular Surgery, Nagano Children’s Hospital, 3100 Toyoshina, Azumino, Nagano, Japan

**Keywords:** Autologous pericardial roll conduit, Non-confluent pulmonary artery, Tetralogy of Fallot

## Abstract

**Background:**

There is great discussion about non-confluent pulmonary artery (PA) reconstruction, and several materials have been used. Autologous pericardium is considered feasible for infectious resistance, autoimmune response, extensibility, and growth potential.

**Case presentation:**

The patient was born at 39 weeks (body mass = 2550 g). He was diagnosed with tetralogy of Fallot, pulmonary atresia, non-confluent PA, and bilateral patent ductus arteriosus. Right and left Blalock-Taussig shunts with patent ductus arteriosus ligations were placed on day 27 and 3 months, respectively. At 19 months (8.8 kg), definitive repair was performed with tricuspid valved conduit concurrent with PA reconstruction using an autologous pericardium roll conduit. The autologous pericardium was treated with glutaraldehyde (autologous pericardium fixed with 0.4% glutaraldehyde for 7 min and rolled as conduit – 12 mm in diameter and 30 mm in length). Following an incision on the visceral side of the PAs before the 1st branch, the autologous pericardial roll conduit was anastomosed. Follow-up angiographies on postoperative months 9 and 57 demonstrated that the PA, including the autologous pericardium roll conduit, had spontaneously enlarged.

**Conclusion:**

Particularly for non-confluent PA, the patients require increased pulmonary beds at an early age because of hypoplastic PA. While size mismatch between the graft and native PA develops as the child grows, size-adjustable extensibility of the PA graft should be noted.

**Electronic supplementary material:**

The online version of this article (10.1186/s13019-019-0913-z) contains supplementary material, which is available to authorized users.

## Background

There is extensive discussion regarding non-confluent pulmonary artery (PA) reconstruction, and several materials have been used [1, 2]. Autologous pericardium is considered reasonable for infectious resistance, autoimmune response, extensibility, and growth potential [3]. Herein, we present a case of tetralogy of Fallot that underwent a definitive procedure concurrent with PA reconstruction using an autologous pericardial roll conduit for non-confluent PA; the case demonstrated an enlarged PA post-procedure.

## Case presentation

The patient (born at 39 weeks; weight = 2550 g) was diagnosed with tetralogy of Fallot, pulmonary atresia, non-confluent PA, and bilateral patent ductus arteriosus. Despite intubation on day 19, desaturation progressed even with prostaglandin administration. Right and left Blalock-Taussig shunts with patent ductus arteriosus were ligated on day 27 and 3 months, respectively. The interrupted pulmonary arterial portion was so long (20 mm) that unifocalization of bilateral PAs was impossible.

**Fig. 1:**
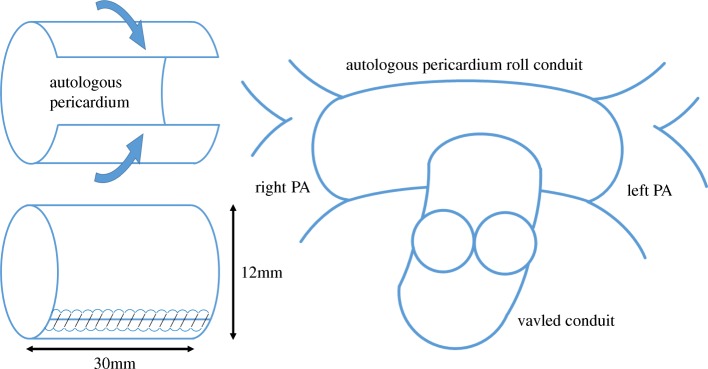
Diagram of pulmonary artery reconstruction. The autologous pericardial roll conduit was anastomosed with the left and right pulmonary arteries. The tricuspid valved conduit was then anastomosed between the right ventricular outlet and the pericardial conduit. PA = pulmonary artery

At age 19 months (8.8 kg), definitive repair was performed with tricuspid valved conduit concurrent with PA reconstruction using an autologous pericardium roll conduit. Cardiopulmonary bypass was achieved, and the previous bilateral Blalock-Taussig shunts were removed. After inducing cardiac arrest, ventricular septal defect was closed with a polytetrafluoroethylene patch. Thereafter, the autologous pericardium was treated with glutaraldehyde (autologous pericardium fixed with 0.4% glutaraldehyde for 7 min and rolled as a conduit – 12 mm in diameter and 30 mm in length). Following an incision on the visceral side of the PAs before the 1st branch, the autologous pericardial roll conduit was anastomosed. Finally, the tricuspid valved conduit was anastomosed between the right ventricular outlet and pericardial conduit (Fig. 1).

**Fig. 2:**
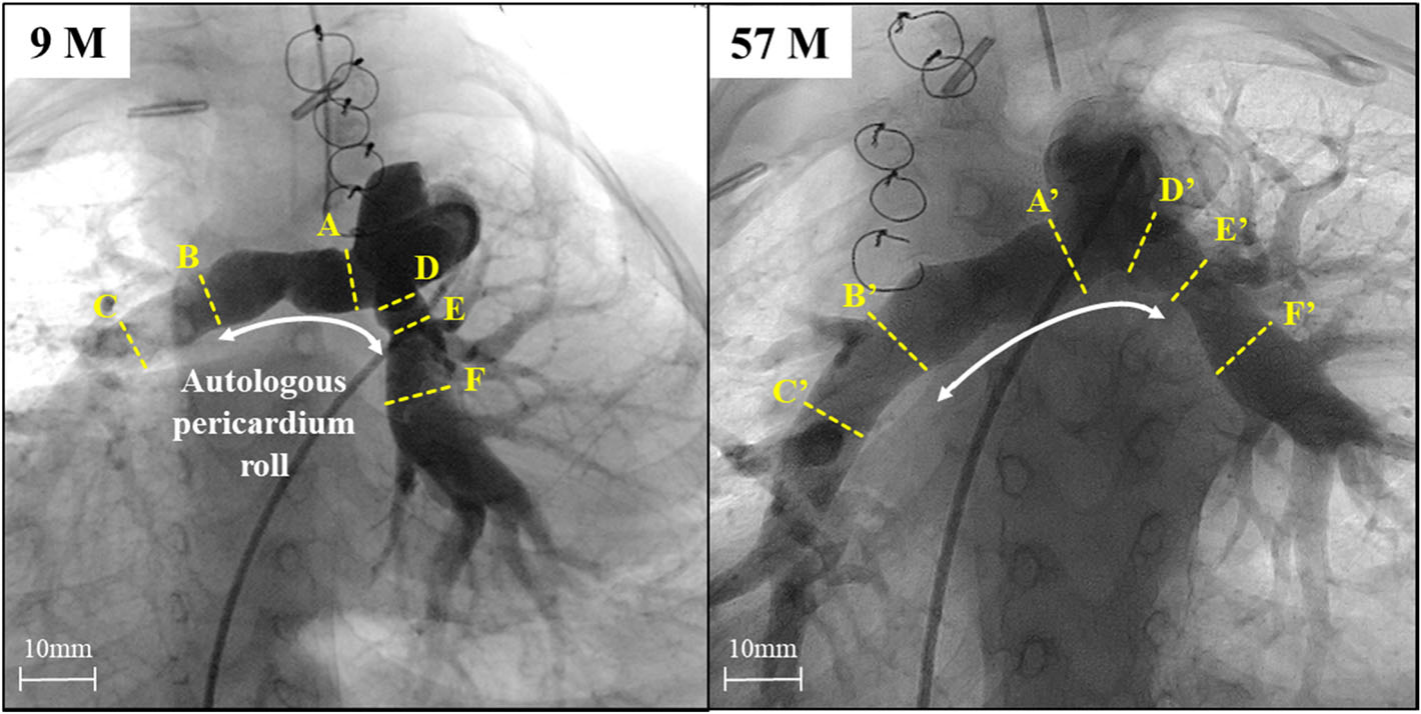
Postoperative pulmonary angiography series. Pulmonary arteries, including autologous pericardium roll, have enlarged. A and A’ = right proximal PA; B and B′ = right distal PA before 1st branch; C and C′ = right basal trunk PA; D and D’ = left proximal PA; E and E’ = left distal PA before 1st branch; F and F′ = left basal trunk PA


The postoperative course was uneventful. The patient was discharged on postoperative day 20. Follow-up angiographies at postoperative months 9 and 57 demonstrated that the PA, including the autologous pericardium roll conduit, had spontaneously enlarged (Additional file 1: Video S1, Table 1 and Fig. 2).

**Table 1:** Follow-up angiography data

	Postoperative 9 mo	Postoperative 57 mo
Diameter (mm)		
Right proximal PA	(A) 8.8	(A’) 13.5
Right distal PA before 1st branch	(B) 8.8	(B′) 14.4
Right basal trunk PA	(C) 8.7	(C′) 10.4
Left proximal PA	(D) 6.8	(D’) 11.3
Left distal PA before 1st branch	(E) 6.8	(E’) 8.3
Left basal trunk PA	(F) 9.1	(F′) 11.9
Blood Pressure, systolic/diastolic (mean) (mmHg)		
Main PA	25 / 7 (14)	21 / 6 (13)
Right PA	17 / 7 (11)	17 / 6 (11)
Left PA	17 / 6 (11)	16 / 7 (11)
PA index (mm^2^/m^2^)	206	330
Rp (WU.m^2^)	2.3	1.9

*PA* pulmonary artery, *Rp* pulmonary resistance, *WU* Wood units

Additional file 1:**Video S1.** Postoperative pulmonary angiography series. Pulmonary arteries, including autologous pericardium roll, have enlarged. (MP4 24477 kb)


## Discussion and conclusions

Autologous pericardium is frequently used in pediatric cardiac surgery. Compared to other materials such as artificial graft [1] and xenopericardia [2], an autologous pericardium is resistant to infection and calcification and free from autoimmune response and has the potential for extensibility and growth. Hibino et al. demonstrated that autologous pericardium could differentiate into the vascular wall, because of the effect of stem cells and the surrounding environment after implantation in the PA [3]. Although we could not confirm the histologic evidence, the enlarged autologous pericardium roll conduit in our case might suggest the growth potential.

Autologous pericardium is often treated with glutaraldehyde. Glutaraldehyde treatment is accessible to technical handling and reinforces the pericardium strength while maintaining its configuration. Non-ringed artificial grafts or xenografts may be compressed by other structures. Lee et al. demonstrated that the most feasible glutaraldehyde treatment method was that with 0.5–0.6% glutaraldehyde for 20 min [4], and indicated the importance of the treatment-associated mechanical properties, degree of fixation, and resistance to enzymatic degradation. Conversely, in our case, glutaraldehyde was more diluted (0.4%) and treatment duration was shorter (7 min) than those in other reports. Our treatment method might suggest the pericardial roll’s extensibility and growth potential. In addition, pulsatile pressure and flow might intermittently expand the pericardial roll, leading to its “adjustable” size as native PA, which accommodates the individual’s body size and age. Continuous low pressure, including Glenn and Fontan circulation, might not affect the size of the pericardium roll conduit.

Particularly for non-confluent PA, the patient requires increased pulmonary beds at an early age because of hypoplastic PA. While size mismatch between the graft and native PA develops as the child grows, size-adjustable extensibility of the PA graft should be noted. Long-term outcomes are unclear; therefore, careful observation is needed in the future.

## Abbreviation

PA: Pulmonary artery
